# Mean platelet volume in patients with prolactinoma

**DOI:** 10.1590/2359-3997000000054

**Published:** 2015-01-01

**Authors:** Abbas Ali Tam, Cafer Kaya, Hüsniye Başer, Reyhan Ersoy, Bekir Çakır

**Affiliations:** 1 Ataturk Training and Research Hospital Department of Endocrinology and Metabolism Ankara Turkey Ataturk Training and Research Hospital, Department of Endocrinology and Metabolism, Ankara, Turkey; 2 Yıldırım Beyazıt University Department of Endocrinology and Metabolism Ankara Turkey Yıldırım Beyazıt University, Department of Endocrinology and Metabolism, Ankara, Turkey

**Keywords:** Prolactin, platelet, mean platelet volume, prolactinoma

## Abstract

**Objective:**

Prolactin is a multifunctional pituitary hormone. The effect of prolactin on platelet activation is not well understood. Prolactinomas are the most common type of pituitary adenomas, and they are medically responsive to dopamine agonists. Mean platelet volume (MPV) is a marker of platelet function and activation. The aim of this study was to evaluate MPV values before and 6 months of cabergoline treatment when normoprolactinemia was achieved.

**Subjects and methods:**

A total of 101 newly diagnosed prolactinoma patients and 102 healthy control subjects were included in the study. Patients with hematological disorders that affect MPV and those on medications were excluded. Prolactin, platelet count and MPV levels were recorded before and 6 months after the initiation of cabergoline treatment (0.5 to 1 mg, two times a week).

**Results:**

There was no significant difference in platelet count and MPV before and after 6 months of treatment with cabergoline in patients with prolactinoma compared with the control group (p > 0.05).

**Conclusion:**

Our results showed that MPV, a marker of platelet function, was unchanged in patients with prolactinoma.

## INTRODUCTION

Prolactin, a hormone that plays roles in onset and maintenance of lactation*,* regulation of mammary gland development, reproduction, osmoregulation, behavior, immune regulation and metabolism, is synthesized by the pituitary lactotrophs (1-3). The role of prolactin in immune system and the regulation of hematopoiesis have been well established (4).

Wallaschofski and cols. have detected short isoform of prolactin receptor in human platelets (5). They also demonstrated that hyperprolactinemia caused increased platelet aggregation via potentiation of adenosine diphosphate (ADP) effects (6). These same investigators have also found increased levels of prolactin in patients with venous thromboembolism, ischemic stroke and transient ischemic attack compared to the healthy subjects (7).

On the other hand, Reuwer and cols. could not have detected the prolactin receptors on the surface of platelets, and subsequently reported that prolactin did not directly modify platelet functions (8). In addition, Wahlberg and cols. found an indirect inhibitory effect of prolactin on platelets in hyperprolactinemic patients, suggesting that prolactin might have a protective role in thromboembolic disease (9).

MPV is reported to be correlated with platelet functions and activation (10). It is an easily measurable parameter and can serve as a useful marker for the documentation of *in vivo* platelet activation (11). In addition, MPV reflects the platelet size. Large platelets are more adhesive and likely to aggregate than small ones (11,12).Prolactinomas are the most common pituitary adenomas and account for approximately 40% of pituitary tumors (13). Typical clinical manifestations of prolactinoma are amenorrhea and galactorrhea in women and impotence, decreased libido and compression findings by the tumor in men. Dopamine agonists, in particular bromocriptine and cabergoline, are the primary treatment in patients with prolactinoma. Administration of bromocriptine and cabergoline normalize prolactin levels and lead to tumor shrinkage. Cabergoline is preferred as the initial treatment of prolactinomas (14).

In this study, our aim was to examine the effect of prolactin on platelet functions by evaluating mean platelet volume before and after dopamine agonist treatment in newly diagnosed prolactinoma patients*. *To our knowledge, our study is the first to address this goal using this method*. *We aimed to provide more evidence on this controversial topic by a retrospective analysis of the medical records of our patients*. *

## SUBJECTS AND METHODS

This study was a retrospective analysis of patients who presented with menstrual irregularities, galactorrhea, erectile dysfunction and diagnosed with prolactinoma at the Endocrinology Department of Ankara Atatürk Training and Research Hospital. All physiological, pharmacological and pathological conditions that could possibly cause hyperprolactinemia were eliminated. Lesions were classified as microadenomas (*≤ *10 mm) or macroadenomas (> 10 mm) according to their size on magnetic resonance imaging of the pituitary gland.

A total of 101 newly diagnosed prolactinoma patients and 102 healthy control subjects were included in the study. Patients taking medications that could affect platelet functions (i.e. anticoagulant drugs, oral contraceptives), suffering from hematological, renal, or hepatic diseases, and with cancer, diabetes mellitus and pregnancy were excluded. All patients received 0.5-1 mg of cabergoline two times per week. The participants’ prolactin, hemoglobin (Hb), hematocrit (Htc) and MPV levels, platelet and white blood cell counts (WBC) before and 6 months after the initiation of cabergoline treatment have been recorded. Venous blood samples had been drawn after 12 hours fasting and blood samples had been collected into tubes containing ethylene diamine tetraacetic acid (EDTA).

This study was approved by the Research Ethics Committee of the institution under protocol No. 117/2014.

Data analysis was carried out using SPSS version 15.0 statistical software. Categorical data were expressed as percentage, and numerical data as mean ± standard deviation. Chi-square test was used for the comparison of categorical variables; Student’s paired and unpaired t-test for the comparison of means. And p values of < 0.05 was considered as statistically significant.

## RESULTS

Of the 101 patients having prolactinoma, 84 were women (83.2%) and 17 were men (16.8 %). There were 82 women (80.4%) and 20 men (19.6%) in the control group. There was no significant difference in gender composition between the two groups (p = 0.60). The mean age was 33.73 ± 11.09 (range, 18 to 60) years in the prolactinoma and 34.04 ± 11.25 (range 18-57) years in the control group, and there was no significant difference in the mean age of the two groups (p = 0.84). No significant difference in platelet count and MPV values was detected between the patient and control groups (p = 0.894 and p = 0.636, respectively). Prolactin levels were higher in the patient group compared to the control (p < 0.001) (Table 1).

**Table 1:** Baseline characteristics of the study groups before cabergoline treatment

	Prolactinoma (101)	Control (102)	p
Age	33.73 ± 11.09	34.04 ± 11.25	0.84
Female	84 (83.2%)	82 (80.4%)	**0.608**
Male	17 (16.8%)	20 (19.6%)	
Prolactin (ng/mL)	117.26 ± 83.52	11.99 ± 5.25	< 0.001
MPV (fL)	9.84 ± 1.34	9.75 ± 1.34	0.636
Platelet count (10^3^/mm^3^)	258.35 ± 49.98	259.38 ± 58.83	0.894
Hb (g/dL)	13.91 ± 0.95	13.77 ± 1.00	0.292
Hct (%)	40.57 ± 2.80	40.42 ± 2.73	0.706
WBC (10^3^/μL)	7.20 ± 1.61	7.26 ± 1.71	0.793

Platelet count and MPV values were evaluated after 6 months of cabergoline treatment when normoprolactinemia was achieved. There was no significant difference between prolactinoma and control groups’ platelet count and MPV values after the 6 month-treatment (p = 0.22 and p = 0.688, respectively) (Table 2).

**Table 2:** Characteristics of the study groups after cabergoline treatment

	Prolactinoma (101)	Control (102)	p
MPV (fL)	10.0 ± 1.53	9.75 ± 1.34	0.688
Platelet count (10^3^/mm^3^)	253.80 ± 53.85	259.38 ± 58.83	0.220
Hb (g/dL)	13.76 ± 1.00	13.77 ± 1.00	0.508
Hct (%)	40.08 ± 4.28	40.42 ± 2.73	0.266
WBC (10^3^/μL)	6.99 ± 1.62	7.26 ± 1.71	0.073

There was no significant difference of MPV, platelet count, Hb, Hct and WBC values in prolactinoma patients before and after treatment (p > 0.05) (Table 3).

**Table 3:** Characteristics of prolactinoma patients before and after 6 months of cabergoline treatment

	Before treatment	After treatment	p
MPV (fL)	9.84 ± 1.34	10.0 ± 1.53	0.239
Platelet count (10^3^/mm^3^)	258.35 ± 49.98	253.80 ± 53.85	0.315
Hb (g/dL)	13.91 ± 0.95	13.76 ± 1.0	0.064
Hct (%)	40.57 ± 2.80	40.08 ± 4.28	0.219
WBC (10^3^/μL)	7.20 ± 1.61	6.99 ± 1.62	0.271

Regarding the size of the tumor, 82 prolactinoma patients (81.2%) had pituitary microadenoma, and 19 (18.8%) had macroadenoma. No significant difference was found between microadenoma and control groups concerning MPV values (p = 0.498). Similarly, there was no significant difference in MPV values between macroadenoma and control groups (p = 0.739). There was no significant relationship between the presence of micro- or macro-adenomas and MPV values.

## DISCUSSION

Prolactin is a multifunctional hormone and its receptors are expressed in almost all organs (15). Dardenne and cols. have investigated prolactin receptor expression in human hematopoietic tissues by flow cytometric analysis and detected prolactin receptor expression in thymus, bone marrow*,* and peripheral blood mononuclear cells (16).

The presence of the prolactin receptor on human platelets is controversial. Wallaschofski and cols. have reported that a short form of prolactin receptors was detected in human platelets (5). Conversely, Reuwer and cols. could not have detected the prolactin receptors on the surface of platelets (8).

Wallaschofski and cols. have published a series of essays about the interaction between prolactin and platelet function. They also demonstrated that increased prolactin level potentiated P*-*selectin expression on platelets and induced platelet aggregation. These same investigators have also found increased levels of prolactin in patients with venous thromboembolism (VTE) without any congenital and acquired thrombotic risk factors for VTE compared to the healthy subjects. They also demonstrated a correlation between prolactin levels and platelet activation in pregnant women and in patients with hyperprolactinaemic pituitary tumors (5,6).

Van Zaane and cols. have reported that venous thrombosis is associated with high prolactin levels (17). On the other hand Reuwer and cols. have reported that prolactin by itself did not activate platelet activation, and not induce platelet aggregation in the *ex vivo* model (8). Atmaca and cols. have shown that although prolactin levels were increased in pregnant women as compared to non-pregnant women, there was no difference in platelet functions (18).

However, Wahlberg and cols. have not confirmed the direct effect of prolactin on platelet activation and aggregation. They suggested that prolactin might affect platelet functions by mechanisms operating over longer exposure time and *in vivo* through indirect mechanisms (9).

MPV has potential prognostic and diagnostic value in hematologic and cardiovascular medicine (19). MPV reflects platelet size and is considered a marker of platelet function. Larger platelets have more granules, produce greater amounts of vasoactive and prothrombotic factors*, *such as thromboxane A2, serotonin and ATP, and aggregate more rapidly (20).

*In vitro* studies for the assessment of platelet aggregation may not correlate with *in vivo* activity of platelets (18). Since platelets are anucleate cells and have little mRNA*,* the capability of platelets to respond to prolactin via protein synthesis is limited (9).

In our study population, we did not demonstrate significant changes in platelet count and MPV values in newly diagnosed prolactinoma patients compared to control subjects. There was no significant difference in platelet count and MPV values after 6 months of treatment with cabergoline. Prolactin had no effect on MPV.

According to the study performed by Erem and cols. there was no significant difference observed in MPV values among patients with prolactinoma compared to the healthy control group. In the same study, while platelet count, fibrinogen, tissue plasminogen activator inhibitor-1 (PAI-1) were increasing, plasma tissue factor pathway inhibitor (TFPI) level was detected decreased. It was suggested that this condition might contribute to an increased risk of atherosclerotic and atherothrombotic complications (21).

Consequently, we have demonstrated that MPV which is a marker of platelet function was not changed in patients with prolactinoma. MPV did not show a significant difference even when normoprolactinemia has been achieved with cabergoline treatment.
